# Iron Homeostasis and Reproduction: Unveiling the Microbiome–Gut–Brain Axis Connection in the Mosquito *Anopheles culicifacies*

**DOI:** 10.3390/cells15151315

**Published:** 2026-07-23

**Authors:** Pooja Yadav, Jyoti Rani, Tanvi Singh, Vaishali Saini, Pooja Rohilla, Vartika Srivastava, Gitanjali Tandon, Nirmala Sankhala, Gunjan Sharma, Suchi Tyagi, Sanjay Tevatiya, Seena Kumari, Rajnikant Dixit

**Affiliations:** 1 Laboratory of Host–Parasite Interaction Studies, Department of Vector Genomics, ICMR—National Institute of Malaria Research, Dwarka, New Delhi 110077, India; yadavpuji60@gmail.com (P.Y.); yadavjyoti712@gmail.com (J.R.); tanvisingh1528@gmail.com (T.S.); vgt1711@gmail.com (V.S.); poojapurva330@gmail.com (P.R.); vartikasrivastava40@gmail.com (V.S.); gitanjali.tandon@gmail.com (G.T.); nirmalasankhala2@gmail.com (N.S.); pachaurigunjan16@gmail.com (G.S.); suchityagi@gmail.com (S.T.); sanjaycena51@gmail.com (S.T.); 2 Academy of Scientific and Innovative Research (AcSIR), Ghaziabad 201002, Uttar Pradesh, India

**Keywords:** mosquito, blood-feeding, iron homeostasis, microbiome, gut–brain axis

## Abstract

Our study investigated how adult female *Anopheles culicifacies* mosquitoes regulate systemic iron homeostasis after blood feeding, a process essential for reproduction, and revealed striking parallels to iron deficiency disorders in mammals. This study identifies that coordinated transcriptional regulation of ferritin and transferrin plays a crucial role in follicle development and egg maturation. Silencing of both genes using ribonucleic acid interference led to severe reproductive impairment, including ovarian arrest in 50% of females, a 40% reduction in oocyte number, and a decrease in first instar larval size. These outcomes correlate with increased reactive oxygen species and altered serotonin receptor expression in the brain, possibly driven by alterations in microbial gut–brain axis communication due to disrupted iron metabolism. In summary, our research provides the first molecular proof and a new conceptual understanding of how iron metabolism disorders may affect microbiome–gut–brain-axis communication and, in turn, reproductive outcomes.

## 1. Introduction

Iron is a universally essential micronutrient, vital for cellular respiration, DNA synthesis, and oxidative metabolism. In mammals, iron homeostasis is tightly regulated to prevent deficiency or overload, with dysregulation contributing to anemia, sepsis, and neuroinflammation [1]. Unlike vertebrates, insects lack hemoglobin-bound iron and instead acquire this metal through dietary sources, including blood meals in hematophagous species such as mosquitoes.

Adult female mosquitoes consume iron-rich blood to initiate vitellogenesis and support oocyte maturation—a process governed by coordinated endocrine and nutrient-responsive pathways [2,3,4,5]. Although the hormonal regulation of vitellogenesis is well characterized, the influence of systemic iron fluctuations on ovarian development and reproductive success remains incompletely understood. Emerging evidence demonstrates a link between nutrient fluctuations, including those associated with blood-derived iron metabolism, and the modulation of broader physiological networks such as the microbiome–gut–brain axis [6]. Because adaptation to blood-feeding is a high-risk, high-reward process, and blood iron serves numerous physiological functions, a multifactor protective mechanism has been proposed to avert oxidative stress in blood-feeding insects [7]. This evolutionary specialization for blood-feeding presents a unique opportunity to investigate the potential mechanism by which adult female mosquitoes regulate systemic iron homeostasis, which may be more akin to mammals [8,9].

The cycling of two iron forms, i.e., reduced ferrous (Fe^2+^) or oxidized ferric (Fe^3+^) ions, between two distinct oxidation states is an important aspect of iron homeostasis in any organism [10]. Unbound ferrous iron can catalyze the production of harmful free radicals via the Fenton reaction, thereby necessitating tight regulation of iron sequestration and trafficking [11]. As a result, the evolution into a protein-bound state of iron limits its accumulation and harmful chemical effects in a living system [12]. In vertebrates, this balance is maintained by proteins such as ferritin (*Fer*) (storage), transferrin (*Trf*) (transport), and hepcidin (systemic regulation) [13]. In hematophagous insects, several studies—particularly those by Oliveira, Oliveira-Paiva, and colleagues—have established important roles for ferritin, transferrin, and heme-detoxifying pathways in managing iron from the blood meal [14,15,16,17]. However, despite this foundational work, the extent to which these proteins interact to coordinate systemic iron mobilization across tissues, or how iron flux integrates with reproductive output and microbiome–gut–brain-axis signaling, remains incompletely understood.

A single blood meal (~2–3 microliters), is sufficient to satisfy the iron need for the gonotrophic cycle in mosquitoes. Export mechanisms for heme and ferritin-bound iron are not yet defined; a mechanistic overview suggests efflux of ferrous ions follows oxidation to ferric ions, which are then bound to hemolymph transferrin for transport to other cells [7]. The absence of the mammalian homolog ferroportin, which exports ferrous ions as a substrate for oxidation via ferroxidase, is still an unaddressed problem in mosquitoes [18]. Although in the tick *Ixodes ricinus*, silencing of intracellular ferritin (fer-2), which also carries a conserved ferroxidase site, and iron-regulatory protein (irp1) significantly impairs reproductive outcome [19], the nature of systemic iron regulation remains understudied.

**Figure 1:**
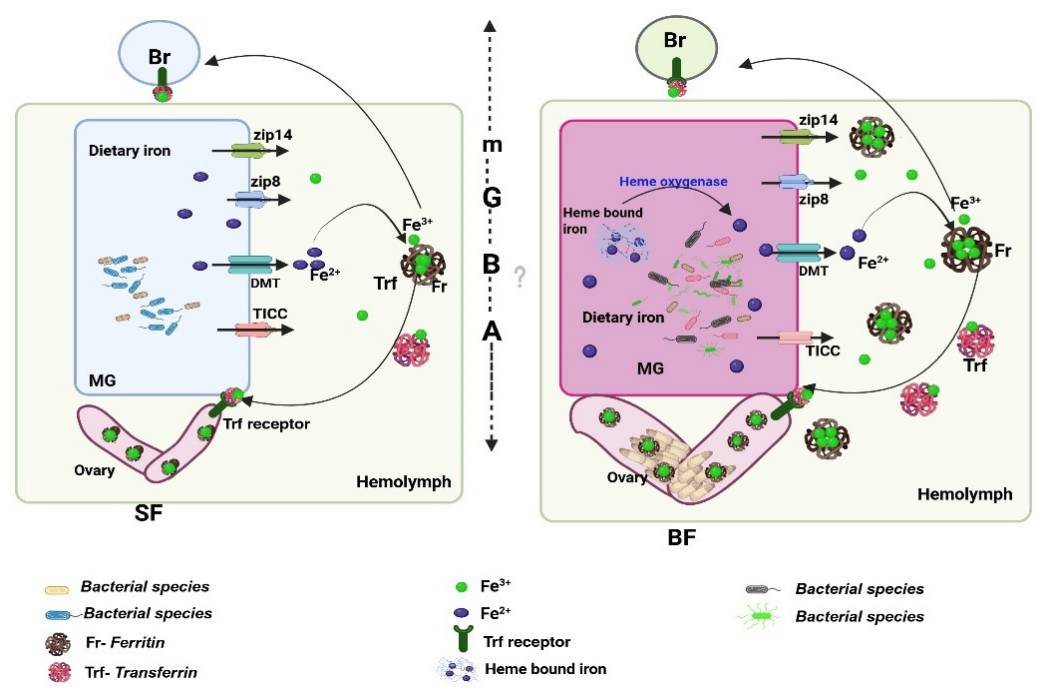
Proposed working hypothesis illustrating the roles of ferritin and transferrin in systemic iron regulation in *An. culicifacies*: Female mosquitoes initially acquire low levels of dietary iron from sugar meals, where free ferrous (Fe^2+^) iron is absorbed across the midgut epithelium via metal transporters such as ZIP family proteins, DMT, and TICC [14]. Within the hemolymph, ferritin oxidizes Fe^2+^ to ferric iron (Fe^3+^) for safe storage, while transferrin transports Fe^3+^ to peripheral tissues, including the brain. Because dietary iron plays a critical role in synapse formation and neural development in the fly [22], it is plausible to propose that iron derived from a blood meal may modulate neuro-signaling processes involved in maintaining physiological homeostasis via the microbiome–gut–brain axis in blood-feeding insects [6]. After an iron-rich blood meal, both ferritin and transferrin are modulated to buffer elevated iron availability and redirect iron toward the fat body and ovaries to support vitellogenesis. The model highlights coordinated, inter-organ iron mobilization across the midgut, hemolymph, fat body, and ovary, illustrating how these proteins maintain systemic iron homeostasis and preserve microbiome–gut–brain axis balance in gravid females.

Previously, we have established that the microbiome–gut–brain axis in *An. culicifacies* integrates metabolic and reproductive signaling in response to blood feeding [6]. This process aids in the optimal digestion and distribution of nutrients essential for ovarian growth, primarily through the fat body and hemolymph [20]. A study of transferrin demonstrated its role in iron transport, and mRNA depletion of this transcript significantly impairs oocyte development in the mosquito *An. culicifacies* [21]. However, the role of the homolog in vertebrates, metalloprotein ferritin, in iron homeostasis and reproductive physiology remains unexplored. Given the established interplay between gut microbiota and host iron metabolism in mammals, we hypothesized that iron homeostasis may serve as a regulatory hub linking reproduction, neurophysiology, and microbial balance in mosquitoes (Figure 1).

To test and evaluate this hypothesis here, we investigated the transcriptional dynamics and physiological functions of ferritin and transferrin in *An. culicifacies*. Using RNAi-mediated knockdown, we uncovered their critical and synergistic roles in promoting egg maturation, mitigating oxidative stress, and maintaining neural and microbial homeostasis. Together, our findings illuminate a central role for iron in coordinating mosquito reproductive biology and reveal a new axis of vulnerability that could be exploited for vector control.

## 2. Methodology

### 2.1. Mosquito Rearing and Maintenance

*An. culicifacies* (sibling species A) was reared and maintained in the ICMR—National Institute of Malaria Research’s central insectary facility under conventional rearing conditions of 28 ± 2 °C temperature, 60–80% relative humidity, and a 12:12 h light/dark cycle [6]. Aquatic larval stages were reared on a powdered mixture of dog food (BIB Pets INDIA Pvt. Ltd., New Delhi, India) and fish food (Tetra, Bawal, India) in a ratio of 60:40, and a small amount was dissolved in the rearing tray containing 500 mL of water. Adult mosquitoes were maintained on 10% sucrose solution supplied via cotton-soaked swabs. All protocols for the cyclic colony of mosquito rearing and maintenance of the mosquito culture were approved by the ethical committee of the ICMR—National Institute of Malaria Research, New Delhi (NIMR/IAEC/2017-1/07).

### 2.2. Exogenous Ferric Chloride Feeding Assay

A control group (100 mosquitoes) of freshly emerged mosquitoes was provided only with a sterile 10% (*w*/*v*) sucrose solution, whereas test groups (100 mosquitoes) received sterile sucrose solution with ferric chloride (Merck, Darmstadt, Germany) at varying concentrations (1 mM, 5 mM, and 10 mM). All solutions were prepared using autoclaved distilled water under aseptic conditions. The sugar solution and the supplemented meal were administered orally through autoclaved sterile cotton swabs. To maintain sterility and prevent microbial contamination, cotton swabs and feeding solutions were replaced at 24 h intervals with freshly prepared sterile solutions. Survival of the mosquitoes was monitored every 24 h of iron supplementation. Hemolymph, fat body, midgut, and ovary were collected from the surviving 20–25 mosquitoes from each set/group (control and supplemented test group) after 48 h of supplementation to assess the transcriptional response of target transcripts. The whole experiment was performed in three independent biological replicates under similar experimental conditions.

### 2.3. Mosquito Tissue Sample Collection

As per the experimental design, the desired tissues were collected from 4-day-old *An. culicifacies* mosquitoes to evaluate spatial–temporal expression. For this purpose, ice-anesthetized mosquitoes (25 mosquitoes) were placed on a slide under the dissecting microscope, and dissected using a sharp needle. Different tissues, including the salivary gland, midgut, fat body, hemolymph, and female reproductive organs, were dissected and pooled separately in Trizol reagent, (Takara, RNAiso Plus, Cat# 9109, Kusatsu, Japan) as described previously [21]. The aquatic development stages, such as egg, larvae, and pupa of the *An. culicifacies* were also pooled separately in Trizol reagent. To explore the fate of iron, we focused on relevant tissues such as the hemolymph, fat body, midgut, and ovary collected from both naive sugar-fed and blood-fed mosquitoes of the same cohort at pre-defined time intervals. The exact number of aquatic stages and mosquitoes used in gene expression data analysis is included at the respective part in the results.

Initially, targeted tissues were collected from sugar-fed mosquitoes aged 3–4 days, while to meet the blood-fed condition, a live and healthy rabbit was placed inside a mosquito cage for blood-feeding. Selected tissues were collected from a fully engorged female mosquito at 0–2 h, 24 h, 48 h, and 72 h after the blood meal for detailed tissue-specific gene expression analysis. For hemolymph collection, an anticoagulant solution containing (60% Schneider’s medium, 10% fetal bovine serum, and 30% citrate buffer) [21] was injected into the thorax, and a small incision was made near the posterior two segments of the distended belly with a minuscule needle, allowing translucent hemolymph to ooze out. Using a micropipette, hemolymph was taken and pooled in Trizol. Fat body was recovered by extracting all abdominal tissues from the last two abdominal segments and then forcibly tapping the abdominal carcass until pale yellow fat body oozed out.

### 2.4. Total RNA Isolation and cDNA Synthesis

Total RNA was isolated from collected samples manually by the Trizol method, as previously described [23]. Isolated RNA was quantified with a Nanodrop 2000 spectrophotometer (Thermo Scientific, Waltham, MA, USA), and ~1 μg of total RNA was used for the synthesis of the first-strand cDNA using the cDNA synthesis kit (PrimeScript^TM^ 1st strand cDNA Synthesis Kit, Cat. # 6110A, Takara, Kusatsu, Japan). Actin was used as a reference gene in conventional PCR to evaluate the quality of the cDNA.

### 2.5. Gene Expression Analysis Through Quantitative PCR

For gene expression analysis, a real-time PCR-based transcriptional profiling assay was performed using the Sybr Green qPCR Mastermix (Takara, TB Green Premix Ex Taq^TM^ II, Cat# RR820A) and CFX 96 Real-Time PCR system (C1000 Touch Thermal Cycler, Bio-Rad, Hercules, CA, USA). The qPCR was performed using three independent biological replicates, each representing samples derived from separate cohorts of mosquitoes. For each biological replicate, two technical replicates were performed to ensure reproducibility and minimize pipetting or instrumental variation. All reactions were carried out under the identical PCR conditions, starting with denaturation at 95 °C for 5 min, followed by 40 cycles of 10 s at 95 °C, 15 s at 52 °C, and 22 s at 72 °C. Each cycle’s fluorescence reading was recorded at 72 °C after it had finished. Two reference genes, namely actin and Rps7, were evaluated (reference gene, primer sequence—Table S4), to select a reliable internal control having stable expression throughout different stages and tissues of the mosquito [24]. The relative quantification was examined using the 2^−ΔΔCt^ method.

### 2.6. dsRNA-Mediated Gene Knockdown Assays in Adult Female Mosquitoes

A double-stranded RNA-mediated gene knockdown assay was executed to suppress target gene mRNA expression. Single-stranded cDNA was first amplified using a dsRNA primer (primer sequence provided in Table S4) with a T-7 overhang primer, as listed in the Table S4. Following amplification, the PCR product was purified through a Gene Jet purification kit (Thermo Scientific, Gene JET PCR Purification Kit, Cat #K0701) and quantified by a nano-drop 2000 spectrophotometer (Thermo Scientific, Waltham, MA, USA). Using the transcript aid T-7 high yield transcription kit (Cat# K044, Ambion, Austin, TX, USA), dsRNA was in-vitro synthesized by employing the purified PCR product as a template. The resulting reaction product was again purified and quantified, and approximately 69 nL (~3 ug/uL) was injected into the thoracic region of a 1–2 day old cold-anesthetized mosquito using a nano-injector (Drummond Scientific, Broomall, PA, USA, Cat# 13681455). Concurrently, for the negative control group, dsRNA of bacterial *GFP* was injected into the mosquitoes of the same cohort. At 48 h post-injection, tissue samples were dissected from three groups i.e., *Fer* only, *Fer* in combination with *Trf*, and control (*GFP*-injected) sugar-fed mosquitoes. To determine the silencing effectiveness, a real-time PCR-based assay was performed as described above.

### 2.7. Assessment of Developmental Effects Following Gene Silencing

To check the developmental effect in the mosquito after disruption of iron homeostasis, we performed an ovary and larval size measurement assay, as described below. Both the control (*GFP* dsRNA-injected) and experimental (*Fer*+*Trf* dsRNA-injected) mosquito groups were fed on the rabbit 48 h post-injection, and only fully fed mosquitoes were selected for further evaluation.

#### 2.7.1. Assessment of Ovarian Development

To evaluate ovary growth, anaesthetized mosquitoes were placed on dissecting slides under a binocular stereomicroscope (Nikon, Toyokawa, Japan), and ovaries were carefully detached in phosphate-buffered saline (PBS) at 72 h post blood feeding. The mature oocytes inside the ovaries were manually counted, and the results were compared to control mosquitoes [21]. However, gradual follicular development was compared among naïve sugar-fed, 24 h, 48 h, and 72 h blood-fed mosquito ovaries. Dissected ovaries from anesthetized mosquitoes were treated with PBS-T (phosphate buffered saline with 0.5% Triton X-100) solution for 2–3 min, followed by thorough washing with 1x PBS. The samples were then incubated with DAPI (4,6-diamidino-2-phenylindole, 300 nM) for 5 min. After staining, excess stain was removed by washing three times with 1x PBS in the dark, and tissue was imaged at excitation/emission: 358 nm/461 nm under a Leica confocal microscope (Thermo-Fisher, Waltham, MA, USA) [25,26].

#### 2.7.2. Larval Size Variation Measurement

After 72 h of a blood meal, mosquitoes were allowed to lay eggs on the moistened paper wrap inside the plastic cup. Following oviposition, the eggs were incubated for 24 h under standard insectary conditions to allow hatching. Post-hatching, individual larvae were immobilized in a small drop of water on a microscopic slide, and the live images were captured using a microscope-mounted digital camera. Measurements were performed using the MagVision image analysis software (MagVision 4.11.20131). For this purpose, images of individual larvae were imported and analyzed under the isogenic conditions of the software. The software automatically calculates dimensions based on pixel-to-micrometer calibration, which accounts for the high precision (two decimal places). The measurement unit were expressed in micrometers (µm). Larval length was determined by digitally drawing a straight measurement line from the anterior tip (head) to the posterior end (tail). For visualization and figure representation, the line thickness and color were manually adjusted, and the scale information was merged with the image. To assess the comparative larval body size change, the relative length of the knockdown larva was compared with the control larva.

### 2.8. Metagenomic Profiling

For WGS-metagenomic analysis, we collected gut samples from 24 h of blood-fed control and *Fer*+*Trf* KD adult female mosquitoes. Before dissection, the body surface of the mosquitoes was sterilized using 70% ethanol for 1 min (five mosquitoes per batch), followed by aeration on a sterilized filter paper. The gut dissection was carried out in 1x PBS, under laminar flow, where the working area as well as the dissecting stereomicroscope were disinfected by using 70% ethanol. We followed a sample pooling strategy, which provides a reliable and comprehensive overview of the gut-bacterial community structure, if the intent is to compare with similar samples [6,27]. For these purposes, pooled guts (50 whole midguts) were collected into a minimal volume (100 uL) of sterile ice-cold 1x PBS and outsourced for WGS sequencing (Shotgun Metagenome, Centyle Biotech Private Limited, New Delhi, India). High-quality genomic DNA was extracted from the collected sample. After isolation, it was quantified and evaluated for quality and integrity. Library construction included appropriate adaptor ligation with DNA fragments, followed by amplification and sequence alignment (https://biologyinsights.com). The raw fastq reads were pre-processed using Fastp v.0.20.1 (parameters: -length_required 50 - qualified_quality_phred 30 - unqualified_percent_limit 30 - average_qual 30) [28]. Read-level taxonomic profiling was done by aligning the processed paired-end reads to the pre-KMA-indexed NCBI 2019 Genome Build (http://doi.org/10.25910/5cc7cd40fca8e) database using KMA [29]. The resulting KMA files were subjected to metagenomic profiling using CCmetagen v.1.2.5 and visualized using krona (CCMetagen-1.2/) [30], and stacked bar plots were plotted using phyloseq R-packages [31]. The relative change in the abundance of the gut-bacterial community structure was examined as described earlier [6].

### 2.9. Oxidative Stress Measurement Assays

To assess the impact of iron homeostasis disruption on oxidative stress-mediated alteration in the midgut and brain, we performed molecular, biochemical, as well as cellular assays as described below.

#### 2.9.1. Molecular Assay

Brains from control and *Fer*+*Trf* knockdown mosquitoes (*n* = 15 per group) were dissected 48 h after dsRNA injection to evaluate the expression of oxidative stress-associated genes. Total RNA was isolated from pooled brain tissues, followed by cDNA synthesis and quantitative real-time PCR analysis of nitric oxide synthase expression in naïve sugar-fed mosquitoes, as described above. For the assessment of superoxide dismutase expression, a separate cohort of control and knockdown mosquitoes was provided a blood meal 72 h post dsRNA injection. Midguts were dissected 24 h post-blood feeding, and superoxide dismutase transcript levels were quantified using qPCR following the same RNA extraction and cDNA preparation workflow.

#### 2.9.2. Cellular Assay

Oxidative stress in naïve sugar-fed (SF), blood-fed (BF) control, and *Fer*+*Trf* KD mosquito groups was evaluated through reactive oxygen species assay. Dissected brain tissue from 15 mosquitoes of each group were incubated with an oxidant-sensitive fluorophore dye CM-H2DCFDA [5-(and-6)-chloromethyl-29,79-dichloro-dichlorofluorescein diacetate, acetyl ester, 2 mM] (Sigma, St. Louis, MO, USA), for 20 min at room temperature under dark conditions. To remove the excess staining, tissues were washed thrice with 1x PBS and observed under a Leica confocal microscope, observing at excitation/emission: 480 nm/530 nm to detect reactive oxygen species accumulation [26].

#### 2.9.3. Biochemical Assay

Alternatively, to estimate the oxidative stress, we collected brains from 15 mosquitoes in 1x PBS, and then briefly spun at 100× *g* for 5 s in a refrigerated centrifuge to extract the supernatant for nitrite quantification using the Griess Reagent Kit (Cat. # G792, Thermo-Fisher Scientific, Waltham, MA, USA). N-(1-aphthyl) ethylenediamine dihydrochloride (Component A) and sulfanilic acid (Component B) were mixed, followed by the addition of 130 μL of the mixture, 20 μL of deionized water, and 150 uL of the test sample as per kit manual. The final mixture was loaded in a 96-well U-shaped ELISA plate and incubated in the dark for 30 min. Furthermore, 20 µL of Griess Reagent and 280 µL of deionized water mixture were considered as a reference sample, which was also loaded onto a plate, and absorbance was recorded at 548 nm using an ELISA Reader (Tecan Unlimited M200pro Instrument, Tecan, Männedorf, Switzerland) [32].

### 2.10. Statistical Analysis

For statistical analysis, test sample data were compared with the control data set using GraphPad Prism software (GraphPad Prism 10.4.1.627), and treatment differences were determined through a one-way ANOVA test for multiple comparisons with respective control, with the help of Dunnett’s multiple comparisons post-hoc test. For pairwise group comparison (control vs. knockdown), an unpaired two-tailed Student’s *t*-test was applied. Statistical significance was established at *p* < 0.05, in addition with 95% CI (confidence interval) for mean differences were calculated to provide an estimate of effect size and the precision of the observed differences. For survival analysis, mortality and survival data of mosquitoes after iron supplementation were analyzed using the Kaplan–Meier method, and survival curves were compared using the Log-rank (Mantel–Cox) test. Hazard ratios with 95% confidence intervals (CI) were calculated to assess the relative risk of mortality between control and treated groups. All experiments were conducted thrice for data validation.

## 3. Result

### 3.1. Fer and Trf Are Co-Regulated for Iron Mobilization in Egg Development

**Figure 2:**
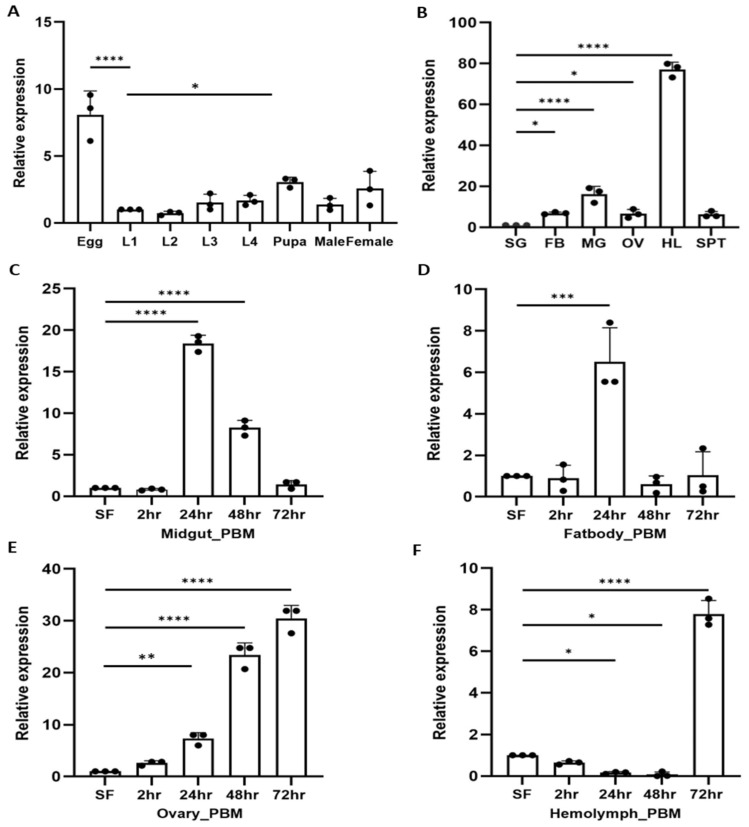
Spatio–temporal regulation of *Fer* expression supports iron mobilization in female mosquitoes: (**A**) Relative expression of *Fer* (ACUA006714) during aquatic development, displaying increased expression of *Fer* in the egg (*p* < 0.0001) and pupa (*p* < 0.0451) of the mosquito. Here, the first instar larva was considered as a control for all test samples (*N* = 3, *n* = 10). (**B**) Tissue-specific expression analysis in naïve adult female mosquitoes exhibits transcript abundance in hemolymph (*p* < 0.0001), midgut (*p* < 0.0001), fat body (*p* < 0.0373), ovary (*p* < 0.0483), and spermatheca in decreasing order, considering salivary gland as a control for all test samples (*N* = 3, *n* = 25). (**C**–**F**) Temporal dynamics of *Fer* expression following blood feeding (PBM). (**C**) Midgut (MG), increased expression at 24 h (*p* < 0.0001), and 48 h (*p* < 0.0001) PBM; (**D**) fat body (FB), up-regulated expression at 24 h PBM (*p* < 0.0001); (**E**) ovary (OV), progressive increase at 24 h (*p* < 0.0025), 48 h (*p* < 0.0001), and 72 h (*p* < 0.0001) PBM; (**F**) hemolymph (HL), peak induction at 72 h PBM (*p* < 0.0001). All the post-blood meal expression assays were performed across three independent biological replicates, and sugar-fed female tissues were considered as a control for all test samples (*N* = 3, *n* = 25) All qPCR data were analyzed using one-way ANOVA with Dunnett’s multiple comparison test. Significance levels: * *p* < 0.05; ** *p* < 0.005; *** *p* < 0.0005; **** *p* < 0.0001. (*N* = number of biological replicates, *n* = number of mosquitoes dissected for sample collection). SG—salivary gland, FB—fat body, MG—midgut, OV—ovary, HL—hemolymph, SPT—spermatheca, PBM—post blood meal.

Structure prediction and modelling analysis predict that *Trf* and *Fer* proteins carry distinct functional domains to bind iron (Supplementary Figure S1). Furthermore, to explore the dynamics of systemic iron regulation, we first characterized the spatial and temporal expression of *Fer* and evaluated the possible correlation with *Trf*. Similar to transferrin [21], we found ~8-fold increased expression of *Fer* in eggs and ~3-fold in pupae among aquatic developmental stages, and ~2.5-fold increased expression in adult females compared to age-matched males (Figure 2A).

Tissue-specific analysis further establishes *Fer* enrichment in the hemolymph and reproductive tissue (Figure 2B and Figure S2A), while *Trf* predominantly localizes to the fat body in the naïve sugar-fed mosquito [21]. Following blood feeding, *Fer* expression surged ~18-fold in the midgut (Figure 2C) and ~6.5-fold in fat body (Figure 2D) at 24 h post-meal, gradually returning to baseline by 72 h post-meal. In contrast, ovarian (Figure 2E) and hemolymph (Figure 2F) *Fer* expression steadily increased ~30-fold and ~7-fold, respectively, over 72 h. Notably, expression of heme oxygenase, a heme-degrading enzyme, was also increased 25-fold at 24 h after blood feeding in the midgut of blood-fed mosquitoes (Supplementary Figure S2B).

### 3.2. Exogenous Iron Elevates Fer Expression and Affects Mosquito Survival

To examine whether iron supplementation perturbs iron homeostasis, we exposed sugar-fed female mosquitoes to increasing concentrations of ferric chloride. At 1 mM, survival was comparable to control; however, concentrations of 5–10 mM significantly reduced lifespan (Supplementary Figure S2C). *Fer* expression in the hemolymph increased proportionally with iron dose (Supplementary Figure S2D), whereas ovarian expression remained unchanged at 1 mM (Supplementary Figure S2E).

### 3.3. Dual Knockdown of Fer and Trf Impairs Reproduction

**Figure 3:**
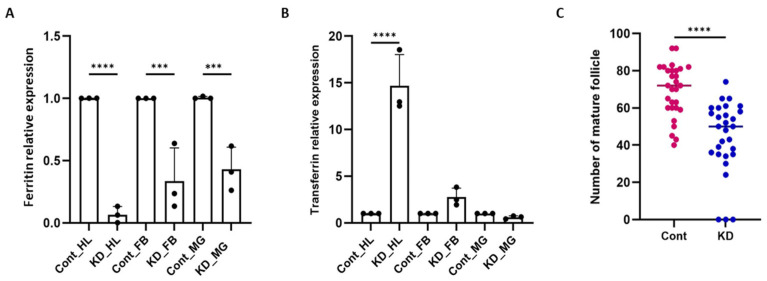
*Fer* knockdown impairs ovarian maturation and modulates *Trf* expression: (**A**) qPCR analysis of *Fer* knockdown efficiency in 1–2-day-old females injected with *dsFer*, using *dsGFP*-injected mosquitoes as controls. *Fer* transcript levels were significantly reduced in hemolymph (HL; *p* < 0.0001), fat body (FB; *p* < 0.0002), and midgut (MG; *p* < 0.0006) (*N* = 3, *n* = 25). Data were analyzed using one-way ANOVA with Šídák’s multiple comparisons test (control vs. knockdown for each tissue). (**B**) *Trf* expression following *Fer* silencing: A significant upregulation of *Trf* was observed in hemolymph (*p* < 0.0001), while changes in fat body (*p* < 0.4021) and midgut (*p* < 0.9823) were not significant (*N* = 3, *n* = 25). Statistical analyses were performed using one-way ANOVA with Šídák’s multiple comparisons test. Significance levels: *** *p* < 0.0005; **** *p* < 0.0001. (**C**) Impact of *Fer* knockdown on ovarian maturation: Dot-plot analysis revealed a significant reduction in the number of mature follicles in *dsFer*-injected females compared with *dsGFP* controls (*p* < 0.0001) (*N* = 3, *n* = 10). Each dot represents the number of mature follicles per female. Data were analyzed using an unpaired two-tailed Student’s *t*-test. (*N* = number of biological replicates, *n* = number of mosquitoes dissected for sample collection). MG—midgut, FB—fat body, HL—hemolymph, Cont—control, KD—knockdown.

To dissect the functional contribution of *Fer* and *Trf* to reproductive physiology, we first performed RNAi-mediated silencing individually and in combination. Silencing of *Fer* alone resulted in approximately 90%, 60%, and 50% reduction of *Fer* transcript in the hemolymph, fat body, and midgut of the injected mosquito group (Figure 3A), respectively, and triggered a ~15 fold compensatory upregulation of *Trf* in the hemolymph (Figure 3B), accompanied by a significant decline in mature oocyte numbers (Figure 3C). A similar compensatory response was observed in the *Trf* silenced mosquito where *Fer* expression increases to ~2.3 fold in ovary (Supplementary Figure S2F).

**Figure 4:**
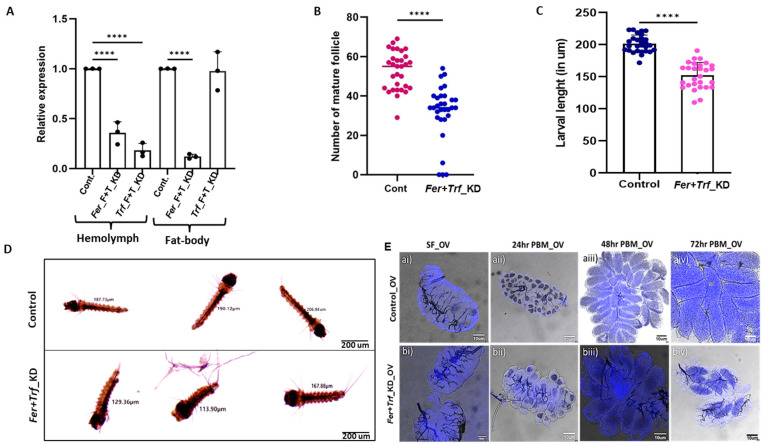
Dual knockdown of *Fer* and *Trf* disrupts ovarian maturation and reduces offspring size: (**A**) qPCR validation of simultaneous knockdown of *Fer* and *Trf* in hemolymph (HL) and fat body (FB) of 2-day-old *dsFer*+*dsTrf*-injected females, showing significant transcript reduction relative to *dsGFP* controls (*p* < 0.0001 for both tissues). Values represent three biological replicates (*N* = 3, *n* = 25). Statistical significance was assessed using one-way ANOVA followed by Šídák’s multiple-comparison test (**** *p* < 0.0001); (**B**) mature ovarian follicle counts demonstrating a marked reduction in follicle number in *dsFer*+*dsTrf* females compared with controls (*N* = 3, *n* = 10 per group). Each dot represents a single ovary pair. Significance determined using an unpaired two-tailed Student’s *t*-test; (**C**) larval body-size measurements showing significantly smaller first-instar larvae derived from *dsFer*+*dsTrf* females relative to control progeny (*N* = 3, *n* = 9). Data analyzed using an unpaired Student’s *t*-test; (**D**) representative micrographs of first-instar larvae from control and double-knockdown females illustrating the reduction in larval length following *Fer*+*Trf* silencing. Images were captured at 4× magnification using a camera-mounted microscope (scale bar = 200 μm), and the size was measured individually through the built-in program available in the image processing software of the camera; (**E**) comparative ovarian development following *Fer*+*Trf* knockdown. Representative DAPI-stained ovaries from control and *Fer*+*Trf* knockdown females under sugar-fed and blood-fed conditions: (ai) sugar-fed controls exhibit compact early-stage follicles with uniform nuclear staining, whereas (bi) *Fer*+*Trf* KD ovaries display smaller, poorly organized follicles with reduced nuclear density; (aii) 24 h PBM controls show normal initiation of vitellogenesis with enlarged, well-organized follicles. In contrast, (bii) 24 h PBM *Fer*+*Trf* KD ovaries exhibit delayed follicle expansion and irregular nuclear distribution; (aiii) 48 h PBM controls reach advanced vitellogenic stages with tightly packed follicles; (biii) KD ovaries remain developmentally delayed, with flattened or irregularly shaped follicles and disrupted epithelial organization; (aiv) 72 h PBM controls display fully matured, elongated ovarioles typical of late vitellogenesis, while (biv) KD ovaries show clear follicular arrest, incomplete growth, and failure to attain terminal maturation stages. All ovaries were dissected, stained with DAPI for 5 min, washed, and imaged using a Leica confocal microscope. Scale bars = 10 μm. (*N* = number of biological replicates; *n* = number of ovaries analyzed per group.) *Fer*—ferritin; *Trf*—transferrin; HL—hemolymph; FB—fat body; PBM—post-blood meal.

Strikingly, co-silencing of *Fer* and *Trf* resulted in decreased expression of *Fer* and *Trf* to 50% and 70%, respectively, in hemolymph (Figure 4A). In stark contrast, dual-silenced mosquitoes exhibited a markedly more severe phenotype: approximately 40% reduction in oocyte number (Figure 4B), and 50% of females exhibited complete ovarian arrest (Figure S3A–C, Supplementary Table S1).

Egg and larval progeny (first instar larvae) of *Fer*+*Trf* knockdown females showed significantly reduced in body size and delayed early developmental progression (Figure S3D; Figure 4C,D, Supplementary Table S2), consistent with impaired maternal iron provisioning during embryogenesis. Microscopic assessment of follicle development revealed disorganized ovarian architecture in double-knockdown females, with many follicles failing to progress beyond early vitellogenic stages, where depletion of *Fer*+*Trf* severely restricts the availability of iron-dependent enzymatic processes required for vitellogenin maturation and proper chorion biogenesis (Figure 4E).

### 3.4. Iron Disruption Reshapes Gut Microbiota Composition and Function

**Figure 5:**
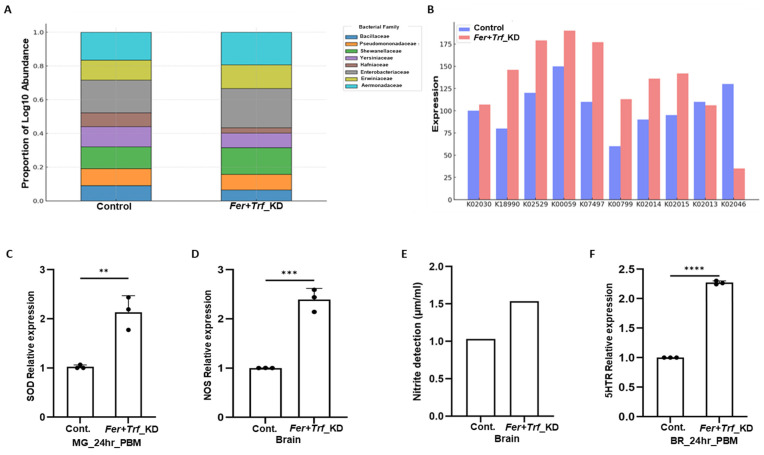
Iron homeostasis disruption alters gut microbiota composition and oxidative stress responses in the brain: (**A**) taxonomic abundance shifts in gut microbiota (Kraken2 analysis): Stacked bar charts display bacterial family-level abundance in control vs. *Fer*+*Trf* KD samples based on Kraken2 classification. The *Y*-axis represents log_10_-transformed relative abundance, with color-coded bacterial families illustrating KD-induced microbiota restructuring. (**B**) Predicted microbial gene expression changes (KMA-based analysis): Bar charts represent differences in predicted metabolic pathway–linked gene expression between control and *Fer*+*Trf* KD samples. Processed paired-end reads were aligned to the NCBI 2019 Genome Build using KMA. Red bars indicate *Fer*+*Trf* KD samples; blue bars indicate controls. (**C**) Relative gene expression analysis of superoxide dismutase (SOD) in midguts of 24 h blood-fed double knockdown (*Fer*+*Trf* KD) females depicts significantly elevated superoxide dismutase expression (*p* < 0.0024) compared with age-matched *dsGFP* controls (*N* = 3, *n* = 25). Data were analyzed using an unpaired two-tailed Student’s *t*-test (** *p* < 0.005; *** *p* < 0.0005; **** *p* < 0.0001). (**D**) Real-time expression analysis of nitric oxide synthase (NOS) in brains of *Fer*+*Trf* KD mosquitoes reveals strong induction of nitric oxide synthase (*p* < 0.0001) relative to *dsGFP* controls (*N* = 3, *n* = 25). (**E**) Nitrite accumulation in the mosquito brain following iron homeostasis disruption. Relative nitrite levels were quantified in control and *Fer*+*Trf* KD females, showing elevated nitrite levels in KD samples. (**F**) Expression of 5HTR in the brains of 24 h blood-fed *Fer*+*Trf* KD females convey significant upregulation (*p* < 0.0001) compared with *dsGFP* controls (*N* = 3, *n* = 25). Statistical analysis was performed using an unpaired two-tailed Student’s *t*-test (** *p* < 0.005; *** *p* < 0.0005; **** *p* < 0.0001). (*N* = number of biological replicates, *n* = number of mosquitoes dissected for sample collection). *Fer*—ferritin, *Trf*—transferrin, PBM—post-blood meal, BR—brain, KD—knockdown.

Given the known links between iron and microbial ecology, we profiled gut microbiota by whole-genome shotgun sequencing. Knockdown of *Fer*+*Trf* was associated with a shift in bacterial composition, characterized by reduced relative abundance of iron-sensitive taxa such as *Aeromonas hydrophila*, *Enterobacter hormaechei*, and *Klebsiella aerogenes*, and increase of *Klebsiella pneumoniae* and *Enterobacter asburiae* (Figure 5A and Figure S4A–D). Functional pathway profiling indicated modulation of enzymes related to iron acquisition, redox balance, and metabolic turnover (Figure 5B and Figure S4E; Supplementary Table S3).

### 3.5. Disruption of Iron Homeostasis Elevates Oxidative Stress and Alters Brain Signaling

To evaluate whether disrupted iron trafficking triggers oxidative stress, we first assessed antioxidant responses in the gut. Superoxide dismutase expression was elevated ~2 fold in the gut of *Fer*+*Trf* knockdown mosquitoes (Figure 5C). In the mosquito brain, oxidative burden was further reflected by an approximately 2.5-fold increase in nitric oxide synthase transcript levels (Figure 5D), and elevated nitrite levels (Figure 5E). As supportive evidence, DCFDA-based fluorescence measurements further showed higher signal intensity in *Fer*+*Trf* knockdown mosquito brains compared with controls (Supplementary Figure S5A–D). Notably, while control mosquitoes displayed a characteristic transient induction of nitric oxide synthase after blood feeding, nitric oxide synthase expression remained unresponsive in the brain of the knockdown female mosquitoes (Supplementary Figure S5E,F). Moreover, there was a remarkable induction of 5-HTR (serotonin receptor) expression in *Fer*+*Trf* knockdown females 24 h post-blood feeding (Figure 5F and Figure S5G).

## 4. Discussion

Our study reveals a coordinated iron-regulatory system involving ferritin and transferrin to balance this trade-off following a blood meal in *An. culicifacies* mosquitoes. Disruption of this regulation impairs reproductive success, alters the gut microbiota, induces oxidative stress, and perturbs brain signaling—collectively pointing to iron homeostasis as a key metabolic activity of mosquito physiology.

After the blood meal acquisition, mosquitoes experience a physiological shift to activate multiple organs to synchronize digestion and reproduction [33]. During the digestion process, the iron released in the midgut via heme oxygenase, a heme-degrading enzyme [34], is then transported to hemolymph, either for storage in the fat body or mobilized to facilitate ovarian development. While testing this proposition, we found temporally and spatially regulated expression of both ferritin and transferrin after blood feeding, suggesting a biphasic model of iron acquisition and distribution. Iron supplementation causes elevated expression of these iron regulatory proteins, which leads to moderate elevations in iron availability, and can also positively influence reproductive output through coordinated regulatory mechanisms [21]. However, high concentrations (5–10 mM) were associated with decreased survival; further studies are needed to determine the underlying mechanisms. Functional silencing of both genes not only arrests follicle development but also reduces egg output and compromises larval fitness, highlighting their role in reproductive investment. The observed phenotypes parallel iron-deficiency-related reproductive disorders in mammals, suggesting evolutionary conservation in iron-dependent fertility regulation [35].

Emerging mammalian studies link iron to microbial ecology [36,37,38], further highlighting the gut microbiota association with iron deficiency-induced anemia (IDA), which may have a significant influence on the absorption of dietary iron [39], although the precise mechanisms remain unclear. Alternatively, in mosquitoes, bidirectional microbiome–gut–brain axis communication is found to be crucial for maintaining the physiological equilibrium and reproductive outcomes [6], but its influence on iron regulation remains undetermined. Integrating with current knowledge, our metagenomic profiling data endorse the idea that iron homeostasis disruption reshapes the gut microbiota, selectively reducing taxa associated with iron acquisition and redox balance, reinforcing the role of iron as a modulator of microbiome–gut–brain axis integrity [40,41]. These alterations in microbial composition and predicted functional pathways indicate a dynamic interplay between host iron regulation and microbial ecology in mosquitoes. While our findings are limited to experimentally induced iron modulation in mosquitoes, the host–microbiome interactions have also been implicated in iron homeostasis in other organisms, including humans [39]. In addition, we observed a significant elevation of reactive oxygen species levels and enhanced nitric oxide synthase activity in the mosquito brain, accompanied by altered expression of serotonin receptors.

In insect brains, reactive oxygen species are not solely indicators of oxidative damage, but also function as modulators of neuronal activity and synaptic plasticity [42,43]. Thus, nitric oxide may act as a key signaling molecule involved in neural communication and behavioral regulation. Our observation on altered reactive oxygen species levels and nitric oxide synthase activity reflects disruption of iron homeostasis, and microbiota modulation may mediate redox-sensitive neural signaling pathways and neurophysiological processes in mosquitoes.

Previous research on the hematophagous bug *Rhodnius prolixus* demonstrated instant increase in 5-HTR level after blood feeding, which then gradually declines with completion of post-blood meal digestion [44]. In mosquitoes, it contributes to gut–microbial interaction, parasite transmission, and ovarian development [45,46]. In mammals, iron deficiency correlated with the expression of 5-HT receptor and serotonin level, which trigger multiple neurotransmission activities, including neuro-behavioral manifestations, neuropsychological disorders, and depression [46,47]. Taken together, our findings indicate that iron status influences neurochemical signaling pathways known for feeding, reproduction, and behavioral disorders via altered serotonin pathways [48].

Together, our findings establish iron homeostasis as a mechanistic bridge between nutrition, neurobiology, microbiota, and reproduction, mirroring the effects observed in mammalian iron deficiency. This work provides a new conceptual framework for understanding the integration of physiological systems in disease vectors and opens novel avenues for targeting iron metabolism as a strategy for mosquito population control.

## 5. Conclusions

Our findings demonstrate a cooperative iron-regulatory axis of ferritin and transferrin, which integrates reproductive physiology, oxidative stress responses, microbial balance, and neurochemical signaling in *An. culicifacies*. Disrupting this axis leads to impaired follicle maturation, reduced offspring fitness, elevated oxidative stress, and altered serotonin-mediated brain signaling, highlighting iron homeostasis as a central determinant of mosquito reproductive success and microbiome–gut–brain axis function.

Notably, the phenotypes observed upon iron disruption parallel several aspects of human iron-deficiency biology, including altered microbial composition, compromised reproductive capacity, and neurochemical imbalance. These cross-kingdom similarities underscore the evolutionary conservation of iron-dependent pathways and reinforce the broader biological relevance of our findings.

While the study provides a comprehensive functional framework, limitations remain—including the use of transient RNAi knockdowns and the species-specific role of the microbes in iron-regulation. Future genetic, biochemical, and single-mosquito microbiome approaches will be essential to refine the mechanistic pathways uncovered here. Overall, this work positions iron metabolism as a convergence point for multiple physiological systems and identifies it as a promising target for next-generation vector-control strategies.

## Data Availability

The metagenomic sequence data have been submitted to the NCBI SRA database under the following accession numbers: SAMN47311708, DNA_AC_BF_MG, and AC_*Fer*+*Trf*_KD_BF_MG, as described in the manuscript. Transcripts, namely, ferritin and transferrin, were identified from pre-existing hemocyte RNA-Seq data submitted to the NCBI with accession numbers AC_HC_SF: SRR12031469 [49].

## Supplementary Materials

The following supporting information can be downloaded at: https://www.mdpi.com/article/10.3390/cells15151315/s1.
