# Supplementary material for: Iron Homeostasis and Reproduction: Unveiling the Microbiome–Gut–Brain Axis Connection in the Mosquito Anopheles culicifacies
Source: Cells. 2026 Jul 23;15(15):1315. doi: 10.3390/cells15151315 (PMC13465019; doi:10.3390/cells15151315)
Supplement: Supplementary file 1 [file cells-15-01315-s001.zip › cells-4148477-supplementary.pdf]

Supplementary data

Iron homeostasis and reproduction: Unveiling the Microbiome-Gut-Brain-Axis connection in the mosquito *Anopheles culicifacies*

Pooja Yadav[1,2], Jyoti Rani[1], Tanvi Singh[1,2], Vaishali Saini[1,2], Pooja Rohilla[1,2], Vartika Srivastava[1,2], Gitanjali Tandon[1], Nirmala Sankhala[1,2], Gunjan Sharma[1,2], Suchi Tyagi[1,2], Seena Kumari[1*], Rajnikant Dixit[1,2*]

[1] Laboratory of Host-Parasite Interaction Studies, Department of Vector Genomics, ICMR-National Institute of Malaria Research, Dwarka, New Delhi, 110077, India.

[2]Academy of Scientific and Innovative Research (AcSIR), Ghaziabad, Uttar Pradesh- 201002.

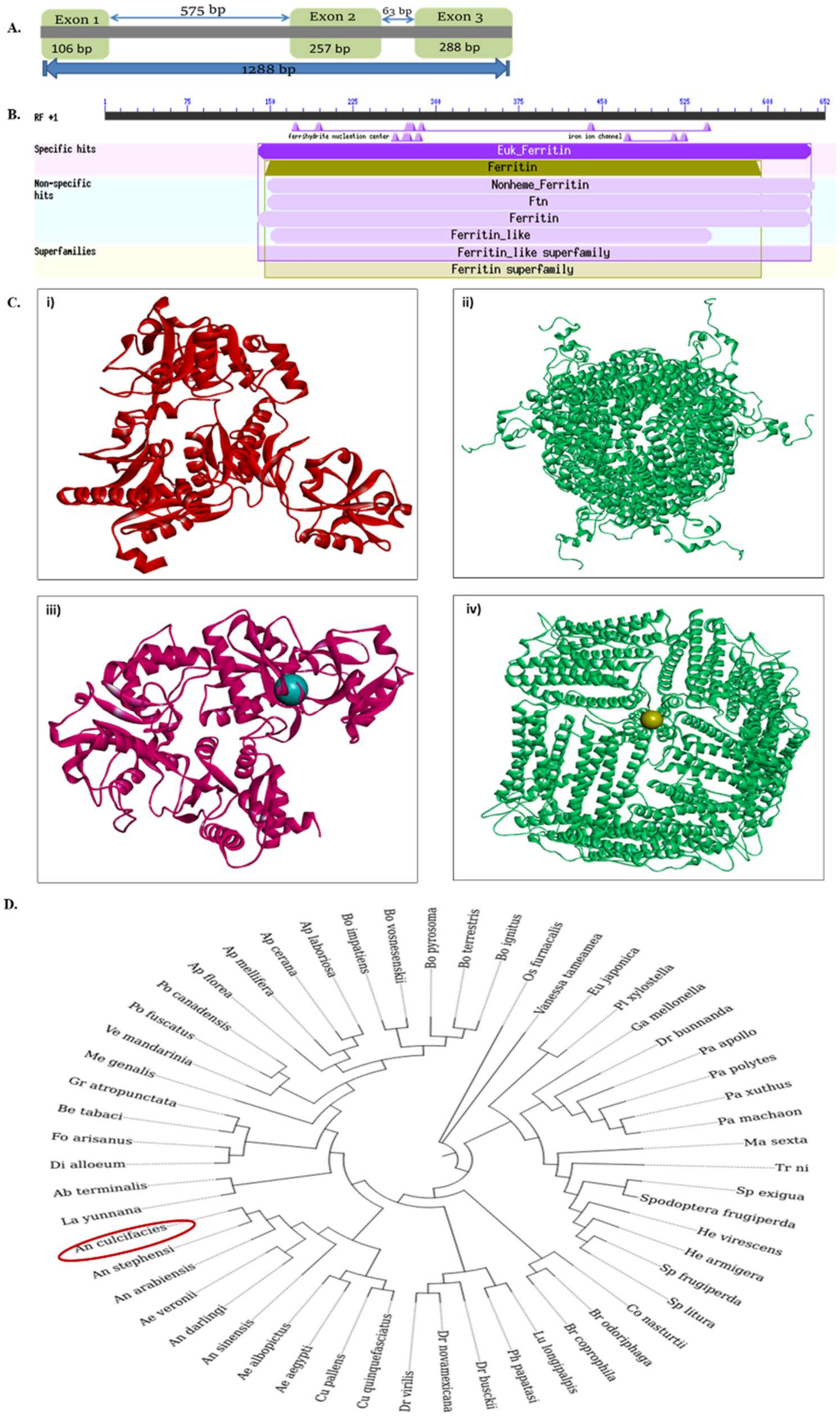

Fig. S1: In-silico analysis and sequence analysis and modelling of *Ferritin* and *Transferrin*. **(A)** Genome organization of *Ferritin* (*Fer*) in *An. culicifacies* (ACUA006714) showing three exons and 2 introns are present in genomic sequence of transcript. **(B)** Domain annotation of *An. culicifacies Ferritin (Fer)*. Detailed in silico analysis was described in previous study of *Transferrin* (Rani et al., 2022). **(C)** **(i)** Final 3D structure of *Transferrin* was constructed using GalaxyHomomer Web Server (http://galaxy.seoklab.org/homomer). **(ii)** 3D model of *Ferritin* was constructed using Modeller version 10.5. Models obtained were further refined and validated with PROCHECK and ModRefiner respectively. After the 3D structures were finalized, active sites of *Ferritin* and *Transferrin* were predicted using CASTp (Computed Atlas of Surface Topography of proteins). **(iii)** *Transferrin* bind with blue round ball indicates ferric (Fe^{3+}) ion while **(iv)** The yellow round ball is ferrous (Fe^{2+}) ion bound to *Ferritin*. Docking of Fe^{2+}(ferrous ion) and Fe^{3+}(ferric ion) done using molecular docking program AutoDock vina 1.5.7. Grid around the packets was created and adjusted

according to the number of points in X, Y, Z-axis so that the entire active site of the proteins is covered. The default value is 0.375 Å between grid points, which is about a quarter of the length of a carbon-carbon single bond. For studying the interaction between the proteins and the ions PyMol and Discovery Studio v. 24.1.0 was used. **(D)** Phylogenetic analysis of selected transcript was done using maximum likelihood algorithm upon 1000 times bootstraps in MEGAX software.

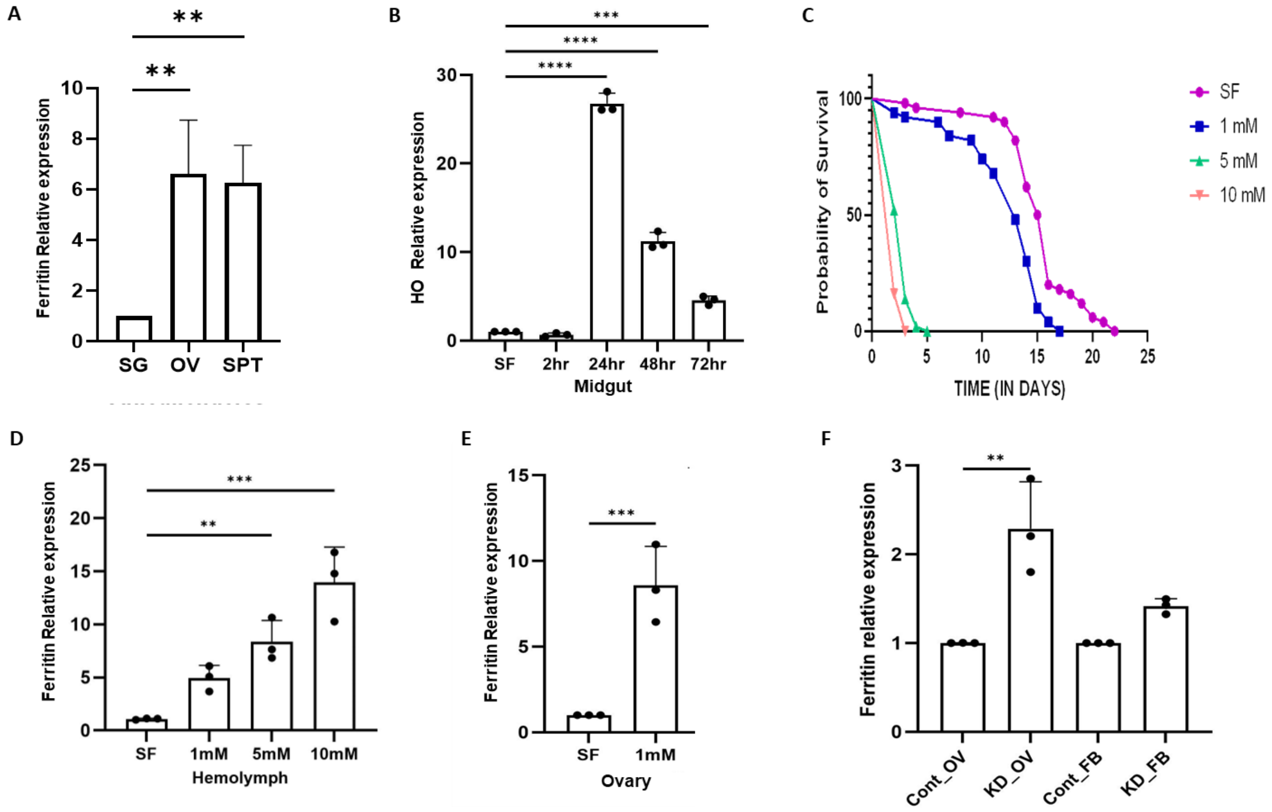

Fig. S2 | Ferritin expression in reproductive tissues, heme oxygenase dynamics, and effects of exogenous iron supplementation in *Anopheles culicifacies*.

(A) *Ferritin expression in reproductive organs.* Relative ferritin transcript levels in female reproductive tissues compared with salivary glands (reference tissue). Data represent three independent biological replicates (N = 3; n = 25 mosquitoes per replicate).

(B) *Heme oxygenase (HO) expression in the midgut following blood feeding.* HO transcript levels in midgut tissues at 24 hr, 48 hr, and 72 hr post–blood meal, relative to sugar-fed controls. Expression peaked at 24 hr ($p < 0.0001$) and declined at 48 hr ($p < 0.0001$) and 72 hr ($p < 0.0004$). Statistical analysis was performed using one-way ANOVA with Dunnett's multiple comparisons test. Significance: *p < 0.05; **p < 0.005; ***p < 0.0005.

(C) *Effect of exogenous iron supplementation on mosquito survival.* Survival of sugar-fed females exposed to increasing concentrations of $FeCl_3$. Higher concentrations (5–10 mM) reduced survival relative to controls (N = 3; n = 25 per condition). Statistical analyses were performed using GraphPad Prism 9.5.

(D–E) *Ferritin expression following exogenous iron supplementation.* (D) Hemolymph ferritin levels increased proportionally with iron dose: 1 mM (ns), 5 mM ($p < 0.006$), and 10 mM ($p < 0.0001$).

(E) Ovarian ferritin expression showed a significant increase at 1 mM ($p < 0.0003$), while higher doses produced non-significant changes. Sugar-fed females were used as controls. Statistical analyses were performed using one-way ANOVA with Dunnett's multiple comparisons test.

(F) *Ferritin expression in Transferrin knockdown mosquitoes.* Ferritin transcripts were significantly upregulated in the ovaries ($p < 0.0008$) but not in the fat body ($p<0.1802$) following Transferrin knockdown. Statistical analyses were conducted using one-way ANOVA with Šídák's multiple comparisons test. For all experiments, N represents biological replicates; n represents the number of mosquitoes used per replicate. SG- Salivary gland, OV- Ovary, SPT- Spermatheca, HO- Hemeoxygenase, FB- Fatbody, KD- Knockdown.

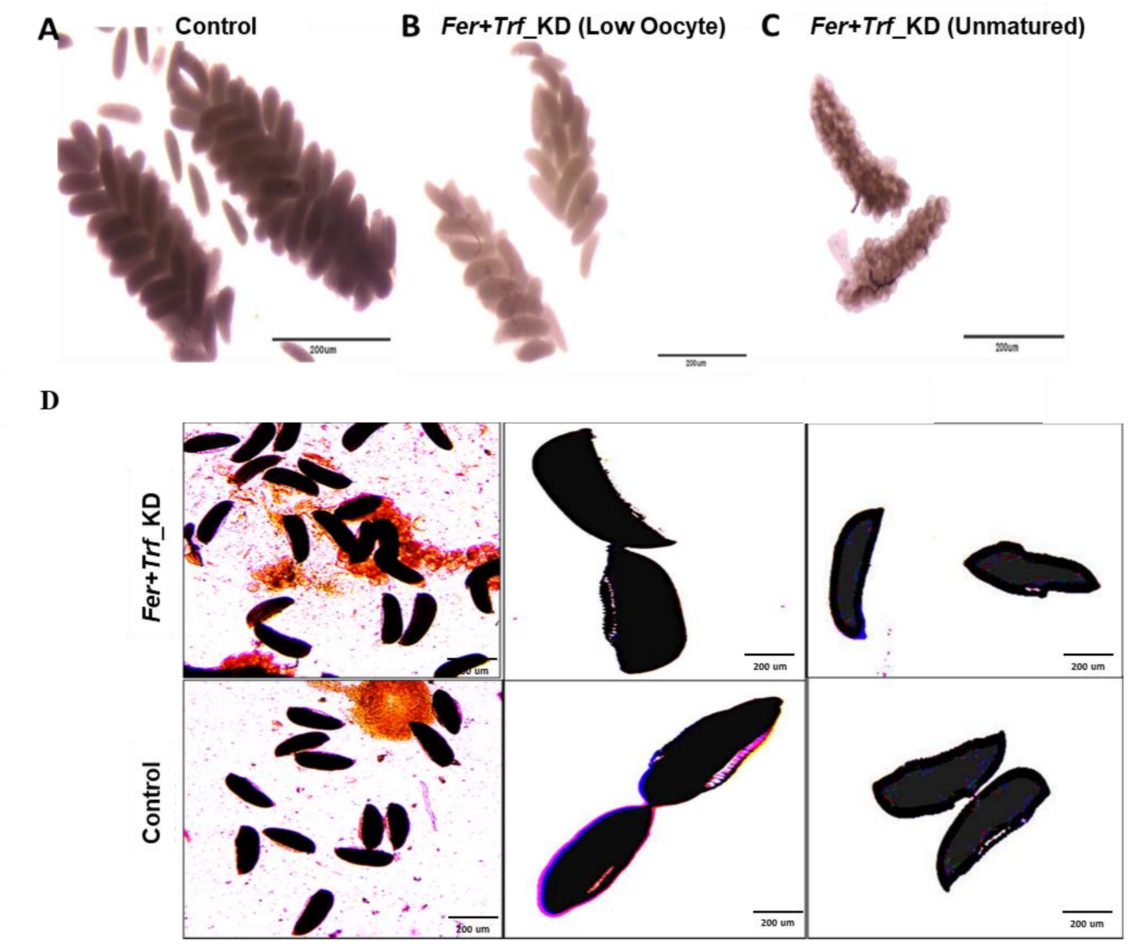

Fig. S3 | Ovarian maturation and egg morphometry in control and Fer+Trf knockdown mosquitoes. (A–C) **Ovarian development at 60 hr post–blood meal:** Ovaries from control (dsGFP) and Fer+Trf knockdown females were dissected at 60 hr post-blood meal and imaged at 4× magnification. (A) Fully matured ovary from a control female showing complete follicular development. (B)Fer+Trf knockdown ovary exhibiting reduced oocyte number. (C) Representative image of an arrested or unmatured ovary in Fer+Trf knockdown females at the same time point. Scale bars were generated in ImageJ.

(D) **Egg morphology variation.** Eggs laid by control and Fer+Trf knockdown females were imaged at 4× magnification using a camera-mounted microscope (Magvision system)(scale bar: 200 µm).

Table S1: Difference in oocyte count between control and Fer+Trf Knockdown mosquito.

S.NO	SET 1		SET 2		SET 3	
	Control	Fer+Trf KD	Control	Fer+Trf KD	Control	Fer+Trf KD
1	51	20	111	70	102	54
2	56	40	88	66	81	60
3	55	29	134	69	94	49
4	69	28	129	73	92	66
5	58	33	112	90	97	51
6	42	40	113	76	76	48
7	43	44	130	80	78	42
8	64	33	119	60	76	66
9	57	38	120	101	74	40
10	43	28	116	68	72	50
11	64	32	125	69	58	11
TOTAL	602	365	1297	822	900	537
		12 ovaries not matured		15 ovaries not matured		13 ovaries not matured

Table S2: Difference in larval length of control as well as Fer+Trf knockdown samples.

S.No.	Length of control larvae (in um)	Length of Fer+Trf KD larvae (in um)
1.	187.73	110.11
2.	198.31	141.07
3.	190.12	139.05
4.	190.53	136.9
5.	205.41	141.59
6.	189.06	141.48
7.	208.13	150.71
8.	196.94	113.9
9.	205.03	133.3
10.	192.46	133.89
11.	189	129.36
12.	203.07	133.79
13.	192.19	133.69
14.	184.04	162.83
15.	192.25	162.13
16.	172.07	170.66
17.	199.2	163.78
18.	198	167.57
19.	199.27	178.67
20.	223.5	162.5

21.	216.52	160.58
22.	219.1	163.96
23.	221.83	167.88
24.	222.01	173.07
25.	209.93	171.72
26.	207.59	178.67
27.	223.61	146.45

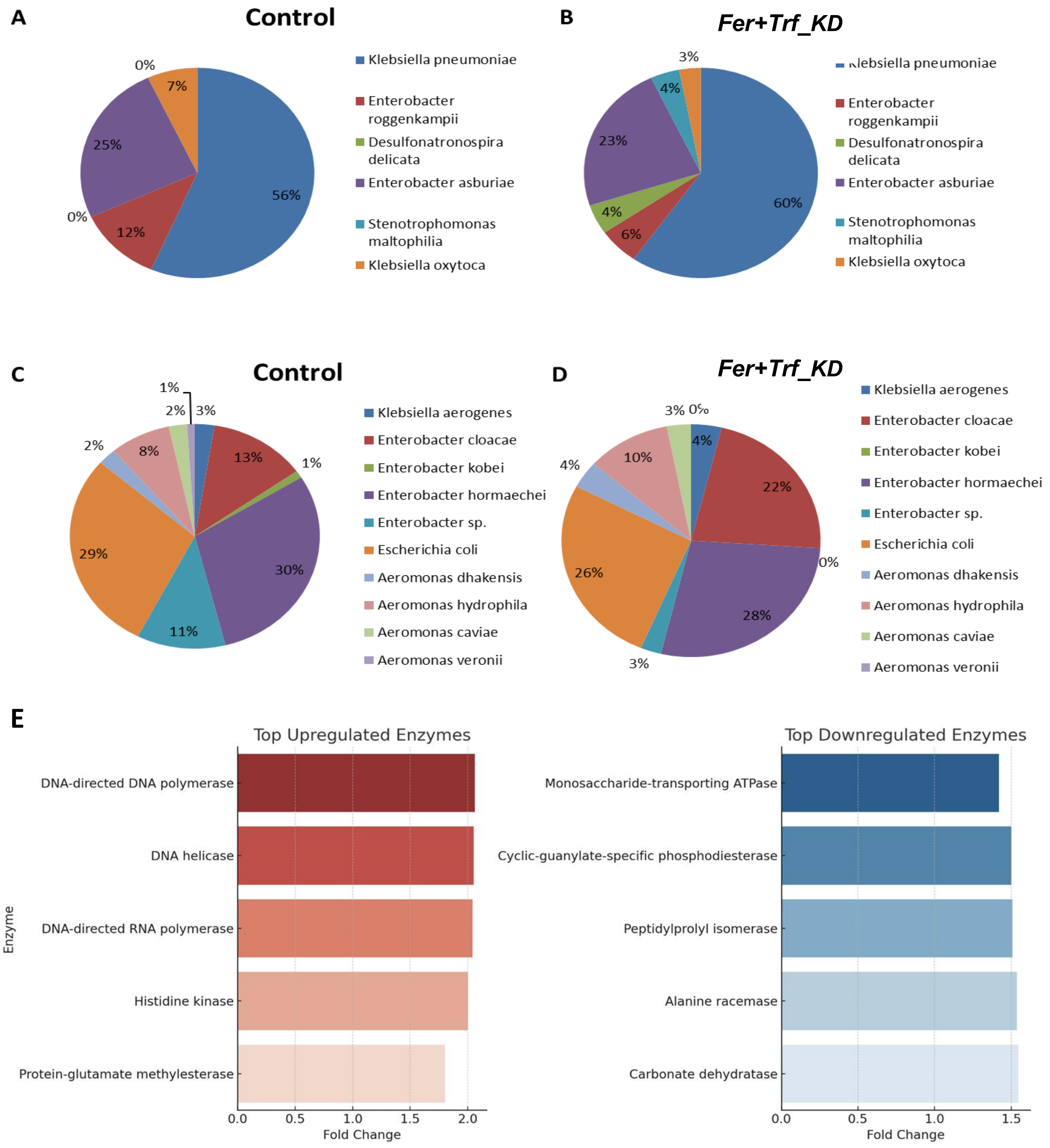

Fig. S4: Bacterial abundance difference in Control and Fer+Trf knockdown samples. Comparative pie chart showing difference in gut bacterial abundance increased after Fer+Trf Knockdown. **(A)** Bacterial abundance in Control **(B)** Fer+Trf Knockdown. Comparative pie chart showing difference in gut bacterial abundance decreased after Fer+Trf Knockdown. **(C)** Bacterial abundance in control **(D)** Fer+Trf Knockdown. The calculated Alpha-diversity included Shanon index (Control: 3.18 and Silenced: 3.25); Beta diversity included Jaccard index (0.32); **(E)** Bakta-Annotated Enzyme Fold Regulation Based on Abundance: Double heatmap showcases the top upregulated (left, red) and downregulated (right, blue) enzymes, annotated using Bakta, based on fold-change regulation in response to Fer+Trf knockdown. Upregulated enzymes (left) show increased abundance in Fer+Trf KD samples compared to Control. Downregulated enzymes (right) exhibit reduced abundance after ferritin knockdown.

Table S3: Gene identified from metagenome sequenecing having role in metabolic pathway and their function.

S.No	Kegg id	Name	Role
**1**	K02030	Bacterial chemotaxis	Cells sense chemical gradients and control autophosphorylation of the Chea histidine kinase
**2**	K18990	Membrane fusion protein	Multidrug efflux system, accessory factors involved in transport
**3**	K00059	Glycosaminoglycan biosynthesis, heparan sulfate backbone	3-oxoacyl-[acyl-carrier protein] reduction
**4**	K07497	Putative transposase	Catalysing movement to another part of a genome,
**5**	K00799	Glutathione s-transferase	Detoxification of exogenous compounds and the relief of oxidative stress.
**6**	K02014	Iron complex outer membrane recepter protein	Bind with substrate and movement towards cytoplasm
**7**	K02013	Iron complex transport system ATP-binding protein	Triggering the entry of the substrate inside the cytoplasm upon catalytic activity of the ABC module
**8**	K02015	Iron complex transport system permease protein	Iron transport through the cell membrane
**9**	K02046	Sulfate/thiosulfate transport system permease protein	Involved in sulfate/thiosulfate import. Probably responsible for the translocation of the substrate across the membrane

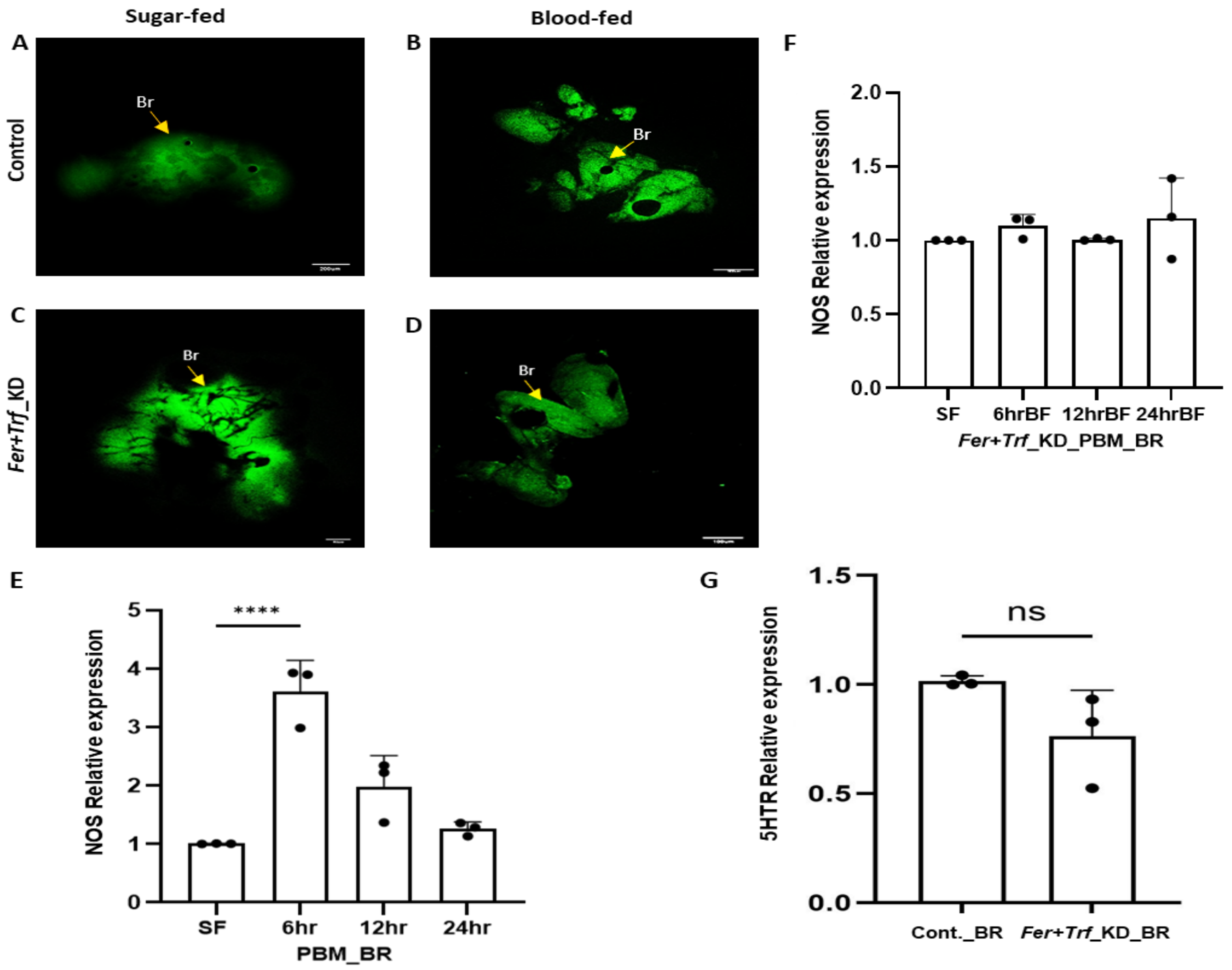

Fig. S5: Effects of Fer+Trf knockdown on brain redox status and neurochemical gene expression. Representative confocal micrographs of CM-H_2DCFDA–stained brains from control and Fer+Trf knockdown mosquitoes under sugar-fed and blood-fed conditions. DCF fluorescence merged with transmitted-light images.

(A) Sugar-fed control brain exhibiting low baseline DCF fluorescence and a narrow intensity distribution. **(B)** 6 hr post-blood meal control brain showing modestly increased DCF signal, consistent with transient post-feeding oxidative activity.

(C) Sugar-fed Fer+Trf knockdown brain displaying elevated and spatially heterogeneous DCF fluorescence, indicative of increased ROS accumulation.

(D) 6 hr post-blood meal Fer+Trf knockdown brain, retaining high ROS signal with broad histogram distribution, suggesting impaired redox regulation following blood ingestion. All micrographs were acquired using a Leica confocal microscope. Scale bar = 200 µm.

(E) NOS expression in control brains showing a significant early transcriptional rise at 6 hr post-blood feeding ($p <$ <0.0001), followed by normalization at 12 hr and 24 hr.

(F) NOS expression in Fer+Trf knockdown brains, demonstrating absence of the typical post-feeding NOS induction, with expression levels remaining comparable to sugar-fed controls.

Gene-expression analyses were performed using three independent biological replicates and evaluated *via* one-way ANOVA with Dunnett's multiple-comparison test; *p < 0.05, **p < 0.005, ***p < 0.0005.

(G) 5-HTR expression in control versus Fer+Trf knockdown brains, showing a slight but nonsignificant reduction in 5-HTR levels in knockdown mosquitoes (p<0.1085).

Expression values represent three biological replicates (n = 25 per replicate, N=3) analyzed using unpaired two-tailed Student's t-test. SF- Sugar fed, BF- Blood fed, KD- Knockdown.

Table S4: List of primer and their sequences.

S. No.	Name of the gene	Left primer	Right primer
1.	Ferritin Heavy chain (*Fer*)	GAAGCTGATCGAATATGCTC	GGTAGTCGACCAGATGGTAA
2.	Transferrin (*Trf*)	AGTTTGAGATAGGCAGCGA	CATCCACCGCATTCATGAA
3.	Fer_DSR	TAATACGACTCACTATAGGGCCTACTTTGCCCAGTACAAG	TAATACGACTCACTATAGGGTAATCGTTGTGATCCTCCTC
4.	Transferrin_DSR	TAATACGACTCACTATAGGGCAGGACCATGACAAGTTCG	TAATACGACTCACTATAGGGCTTGCGGCGCTTCTTTAT
5.	NOS	ATGAGGACCAACTATCGGG	GCCTTGGTGACAATGCTC
6.	SOD3	TAGAAGGTTTACGACCAGGA	ATGATGTTACCGAGATCACC
7.	Hemeoxygenase 1	GCGCAAAATAGTAGACGAGT	AACTGCGTTAAAACGATACC
8.	5HTR	ATGATCTCGCGTAACTCCTC	ATCGGATTGACCAGACTGC
9.	16S	TTGGAGAGTTTGATCCTGGCTC	ACGTCATCCCCACCTTCCTC
10.	Ac_ACT	GCGGTATACTGACACTCAA	CAAACATGATCTGTGTCATC
11.	Ac_RSP7	ATCGCTATGGTGTTCGGTTC	TTGTTGAACTCGACCTCACG
